# Dental anxiety in first- and final-year Indian dental students

**DOI:** 10.1038/s41405-019-0017-9

**Published:** 2019-10-16

**Authors:** Chitta Ranjan Chowdhury, Shahnawaz Khijmatgar, Avidyuti Chowdhury, Stewart Harding, Edward Lynch, Martin Gootveld

**Affiliations:** 1 Department of Oral Biology & Genomic Studies, AB Shetty Memorial Institute of Dental Sciences, Nitte Deemed University, Mangalore, 575018 India; 2 Academic Dean, University of Bolton City of London Dental School, Bolton, UK; 3 Global Child Dental Health Task force, Kings College London, London, UK; 4 Dean, University of Bolton City of London Dental School, Bolton, UK; 5 Biomedical and Clinical Research, School of Dental Medicine, University of Nevada, Las Vegas, USA; 6 Institute of Health and Life Sciences, Health and Life Science, De Montfort University, The Gateway, Leicester, LE1 9BH UK

**Keywords:** Oral-health-related quality of life, Dental anxiety and phobia

## Abstract

**Objectives:**

The study aims to investigate dental anxiety in first- and final-year undergraduate dental students in India.

**Design:**

Questionnaire Study Setting: BDS Students in four University dental colleges in India carried-out during 2013 and 2014.

**Subjects (materials and methods):**

The students (*n* = 614) were assessed using a pre-tested questionnaire. We estimated the level of dental anxiety by using the Modified-Dental-Anxiety-Scale (MDAS). ANCOVA and Mann–Whitney U, and Chi-squared contingency tests were employed to analyze the extensive dataset acquired. Univariate clustering analysis and principal component regression were also applied. Students had similar demographic and lifestyle patterns.

**Interventions:**

Assessments of the level of dental anxiety amongst undergraduate dental students.

**Main outcome measures:**

Mean ± SD MDAS scores for first- and final-year Bachelor of Dental Surgery (BDS) students were 12.96 ± 4.00 and 10.54 ± 3.41, respectively.

**Results:**

Six hundred and fourteen (*n* = 614) students from four dental colleges were included in this study. In total 77% were female (*n* = 478) and 23% were male (*n* = 136). The mean age of the first- and final-year students were 18.31 and 21.54 years, respectively. First-year BDS students had dental anxiety score (Mean ± SD 12.96 ± 4.00) compared to that of the final year (10.54 ± 3.41), a difference which was very highly statistically significant (*p* < 0.0001).

**Conclusion(s):**

Dental anxiety was moderately higher amongst first year BDS students over that of final-year students but it is lesser than the dental phobic threshold level.

## Introduction

Dental anxiety is often reported worldwide. The prevalence of dental anxiety among the general population is between 3.9 and 11%.^1^ Dental anxiety is a psychological feeling, which can partially or completely prevent a person from receiving dental treatment. It prevails in all age groups, but is most commonly observed among young adults between 18 and 26 years of age.^2–4^ However, the prevalence rate among dental students is lower when compared to that of the general population.^3,4^

Dental anxiety leads to negative attitudes towards receiving dental treatment; consequently, these students live with dental problems for prolonged periods.^5–7^ Indeed anxious dental patients, including dental students themselves, suffer more from dental diseases, simply because they cancel or delay their visit to a dentist, and consequently complicate their disease condition.^7,8^ Therefore, a dentally anxious student is more unlikely to be able to maintain a good quality of life, in view of their altered oral health status.^9^

There are several theories which attempt to explain the causes of dental anxiety. Of these, unpleasant experiences whilst receiving dental treatment from a non-empathetic or ‘bullying’ dentist appear to represent the primary cause of dental anxiety.^10,11^ There are also specific stimuli such as the observation of needles for local anesthetic injections, dental instruments, the characteristic odor of a dental clinic, the use of rubber-dams, together with noises from dental drills, which may all trigger dental anxiety, either singly, or two or more synchronously in concern.^6,12^ Nevertheless, not every patient who has experienced one or more painful dental procedures suffers from dental anxiety. Its etiology is therefore likely to be multi-factorial, which largely includes experience of a previous painful treatment combined with the particular personality traits of affected patients.^13–17^ In this context, some of dental (BDS) students fall into this category. Previous studies have shown that a significant number of BDS students have a considerable amount of anxiety of receiving dental treatments.^18–21^ However, effective communication has promoted the induction of a positive attitude towards dental treatment in both Bachelor of Dental Surgery (BDS) students and oral health professionals in general.^22,23^ Indeed, dental professionals play a crucial role in the management of dental anxiety.^22,23^ Therefore, an understanding of the level of dental anxiety amongst dental students will provide a platform to work from in order to explore if, and how exactly, this phenomenon significantly impacts on the abilities of future cohorts of dental professionals to effectively manage their patients. Also notable is the conceivable hypothesis that anxiety experienced by dental students is associated with oral health, poor dietary habits or indeed a higher body mass index (BMI).^24–26^

Hence, the aims of this study were to investigate the level of dental anxiety in BDS students, and also assess some possible inter-relationships with other variables such as age and gender, the former strongly correlated with student study year. Overall, the investigation was designed to determine if there are any significant differences in dental anxiety between first- and final-year BDS students.

## Materials and methods

The study received ethical approval from the Central Ethics and Research Committee of Nitte University. Data collection was performed during 2013 and 2014. The sample population was designed to include students from four dental schools located in the state of Karnataka, India. These dental institutes included A.B.Shetty Memorial Institute of Dental Sciences (ABSMIDS) of Nitte; Yenepoya Dental College (YDC), Yenepoya University; Manipal College of Dental Sciences (MCODS) of Manipal University; and A. J. Shetty Institute of Dental Sciences (AJSIDS), Rajiv Gandhi University of Health Sciences, Mangalore, Karnataka, India. The cohort group can be considered as a representative sample of its category for the population of India.

Dental anxiety level, along with related information was collected from all participants recruited. The original English version of the Modified Dental Anxiety Scale (MDAS, Table 1) was employed,^27,28^ which is a five-item questionnaire containing questions about respondents’ level of anxiety whilst visiting a dental surgeon, with a response scale ranging from 1 (not anxious) to 5 (extremely anxious). The total scores ranged from 5 to 25. A score <11 is considered normal, whereas those lying between 11 and 18 represent moderate anxiety. Scores >19 represents extreme anxiety. The reliability of the English language version of the MDAS questionnaire had an internal consistency of 0.89, and a test-retest value of 0.82. All the students were able to understand the English version of MDAS. The BDS course in the 4 university participant sources is in the English language, and all the BDS students in these cohort groups speak, read and write English competently.

**Table 1:** List of Modified Dental Anxiety Scale (MDAS) anxiety questionnaire

Items	Modified dental anxiety scale (MDAS) anxiety questionnaire	
1	If you went to your dentist tomorrow, how would you feel?	1. Not anxious
2	If you were sitting in the waiting room, how would you feel?	2. Slightly anxious
3	If you were about to have a tooth drilled, how would you feel?	3. Fairly anxious
4	If you were about to have your teeth scaled and polished, how would you feel?	4. Very anxious
5	If you were about to receive a local anesthetic injection in your gum, how would you feel?	5. Extremely anxious

### Questionnaire survey

In order to avoid any response bias, students were not provided with any prior information regarding dental anxiety prior to commencing the study. The students were given 15 min to answer a pre-printed questionnaire (Table 1). They were fully informed about the voluntary nature of their participation and their rights as volunteers to withdraw from this study at any point in time during its course. Participant questionnaire respondents were invigilated in order to ensure non-collusion and lateral discussion, and any form of cheating was prevented during participant completion of the questionnaire.

Questions related to oral hygiene frequency of dental visits, periodontal problems, and preference of dentistry as a career was included in the questionnaire. The students were provided with the opportunity to request any further clarification.

### Statistical analysis

**Fig. 1:**
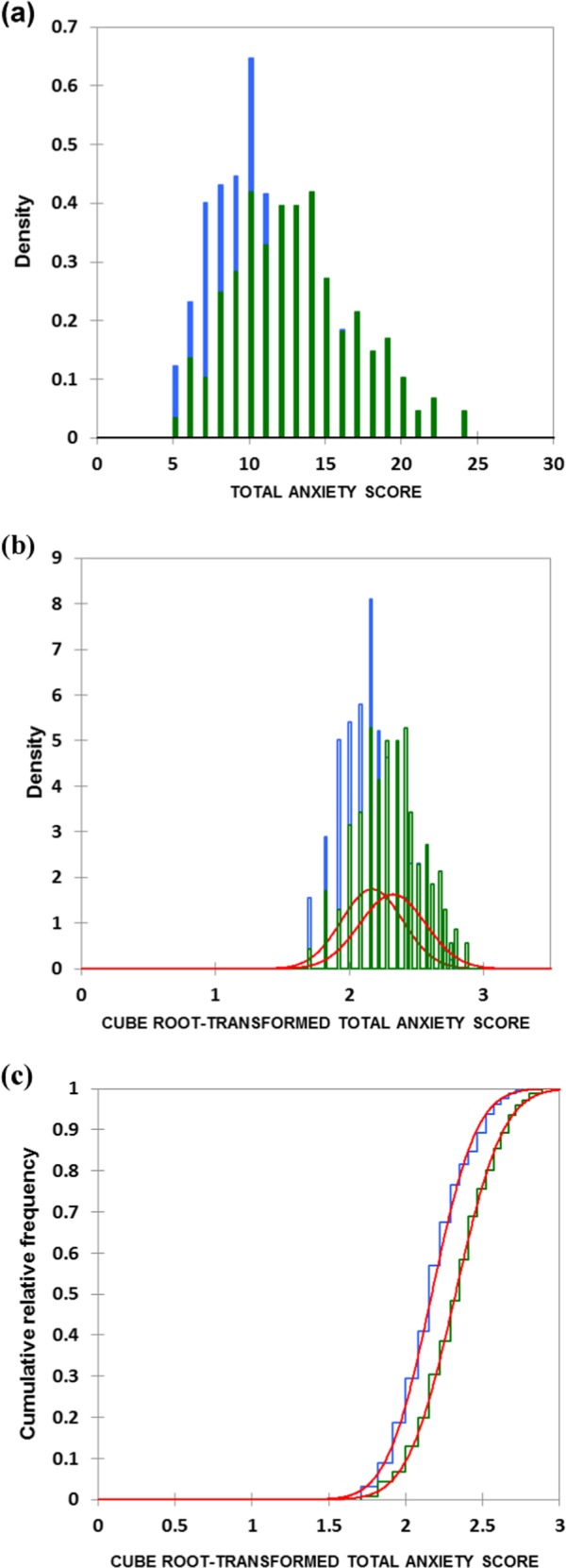
Normality distribution density plots of (**a**) the untransformed (raw data) total dental anxiety scores for first- and final-year BDS students (green and blue respectively), and (**b**) following cube root transformation of these datasets. Fitted normal distributions are depicted in red in (**b**). **c** Plots of cumulative relative frequency against the cube root-transformed total dental anxiety scores for first- and final-year BDS students (blue and green respectively), demonstrating an excellent fit to a normal distribution curves (red) for both groups

Tests for normality of the total anxiety score values for both student year classifications were performed by the Jarque–Bera test. Evaluations of variance heterogeneity between these two student group classifications were performed by the F variance ratio test. For the raw (untransformed) dataset, the distributions of the total anxiety scores for both groups demonstrated strong departures from normality (*p* *=* 0.004 and <10^−4^ for the first- and final-years respectively), and therefore data were transformed to their cube root values (Fig. 1). A further data transformation applied was the Box–Cox transformation. These transformations were further justified, since there was an extremely highly significant difference found between the variances of these groups (F-test), i.e., that for first-year students was much greater than that for the final-year cohort (*p* *=* 0.004).

Application of these cube root- and Box-Cox-transformations effectively normalized both first- and final-year datasets (*p* *=* 0.278 and 0.148 respectively for the former transformation, and 0.181 and 0.242 respectively for the latter one). We therefore elected to perform the ANOVA and ANCOVA (models 1 and 2 respectively) analyses on the cube root-transformed dataset described below. Moreover, these analyses were supported by that employing the direct Mann-Whitney non-parametric U test for differences between the total anxiety scores of these two student groups without consideration of the gender and age variables, which were both found to be insignificant ‘within-BDS student year’ groups via the ANCOVA analysis model 2 applied, as described below. This non-parametric test employed 10,000 Monte-Carlo simulations to compute its *p* value.

Analysis-of-variance (ANOVA) was employed in model 1 in order to test for significant differences between the total dental anxiety parameter (y_*ijk*_) between BDS study years, and ANCOVA was applied in model 2 to investigate the influence of genders and student ages ‘nested’ within these study years. The first of these is described by the mathematical model given in Eq. 1, where Y_*i*_, G_*j*_ and YG_*ij*_ represent the student year, gender and student year x gender interaction sources of variance respectively (all fixed effects), µ the value of y_*ijk*_ in the absence of all these effects, and e_*ijk*_ representing fundamental error.

1 $${\mathrm{y}}_{ijk} = {\mathrm{\mu }} + {\mathrm{Y}}_i + {\mathrm{G}}_j + {\mathrm{YG}}_{ij} + {\mathrm{e}}_{ijk}$$

**Fig. 2:**
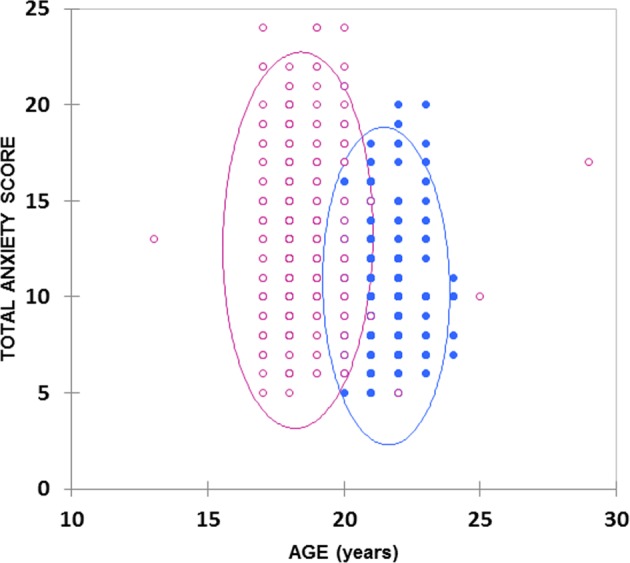
Plot of total anxiety score *versus* age for first- and final-year Indian dental students (pink and blue respectively). The smaller number of datapoints visible than the total student participant sample size (*n* = 614) visible arises from their manifold level of overlap in the diagram. 95% confidence ellipses for both sample groups are also indicated

Since there was a strong positive correlation between year of study and student age (Fig. 2), the latter variable was primarily removed from the ANOVA model 1 employed. However, following the establishment of a clear significant difference between the two study year classifications, ANCOVA was also then applied to test for differences ‘between-ages’ within each of the two study years explored (Eq. 2), in which A_*j*_ and GA_*ij*_ represent the age covariable and its interaction with the gender source of variation (i.e., gender × age interaction effect).

2 $${\mathrm{y}}_{ijk} = {\mathrm{\mu }} + {\mathrm{G}}_j + {\mathrm{A}}_J + {\mathrm{GA}}_{ij} + {\mathrm{e}}_{ijk}$$

Chi-squared contingency table analysis was conducted in order to determine any associations between the numbers of male and female students present in each year of study group, and this involved 5000 Monte-Carlo simulations and a standard bootstrap interval of 100 samples.

Principal component regression (PCR) analysis was employed to investigate the relationship between principal components formed from (1) strongly correlated student year and age variables, and (2) genders alone. This model involved a minimum consideration of 80% of the total variance, and a tolerance of 10^−4^. Total anxiety score values and participant ages were standardized (i.e., subtraction of their total variable mean values followed by division of their sample standard deviations so that they both had means of 0 and unit variances) prior to PCR analysis.

All statistical analyses, including two-sample *t*-tests, were performed using *XLSTAT2014* and *SPSS* software.

## Results

For this study, all 614 students requested to participate responded to the questionnaire, i.e., a 100% response rate. The age of the sampled population ranged between 18 and 22 years, who were studying within the first- and final-years of their undergraduate BDS course. Mean ± 95% confidence intervals (CIs) for the ages of the first- and final-year student participants recruited to the investigation were 18.31 ± 0.135 and 21.54 ± 0.116 years. This difference in mean ages was highly statistically significant (*p* *<* 10^−4^), as expected (two-sample *t*-test). The gender-wise distribution of the students was 136 (22.1%) male, and 479 (77.8%) female.

The male-to-female (M:F) ratio of BDS students in India is ~20:80, and this index is common throughout this country, including Karnataka state. This is likely to be explicable to the students’ parents/guardians preference for their daughters to be dental or medical clinicians. Moreover, the study of dentistry in India represents the second-most popular choice of many female students who are not accepted to study medicine.

There was no significant difference between the mean ages of groups classified by gender i.e., 19.68 and 19.69 years for males and females respectively (two-sample *t*-test).

A plot of total dental anxiety score *versus* age (Fig. 2) revealed clearly distinguishable clusterings for the first- and final-rear BDS students. Mean ± SD total anxiety score values for the first- and final-year students were 12.96 ± 4.00 and 10.54 ± 3.41 respectively (95% confidence intervals 12.54–13.38 and 10.12–10.93, respectively).

**Fig. 3:**
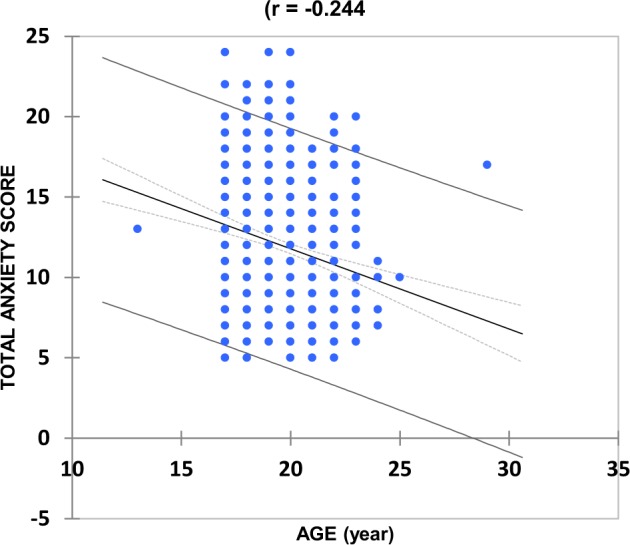
Overall plot of total anxiety score *versus* student age for the combined first- and final-year BDS students (*r* = −0.244). Although the negative correlation coefficient observed is low, this relationship was very highly statistically significant (*p* < 10^−4^) in view of the very large sample size (*n* = 614), and demonstrates an age-dependent decrease in the total anxiety score. 95% Confidence intervals for both the mean regression values and individual observations are provided

The critical importance of the study year variable was also demonstrated by linear regression analysis plots of untransformed total anxiety score against age, which was extremely statistically significant for the entire total anxiety score response dataset (Fig. 3), i.e., that comprising the first- and final-year students combined (*r* = −0.24, *p* *<* 10^−4^). However, this relationship was not at all significant when these plots were performed for each study year separately (*r* = 0.040 and −0.039 only for first- and final-year BDS students respectively).

**Fig. 4:**
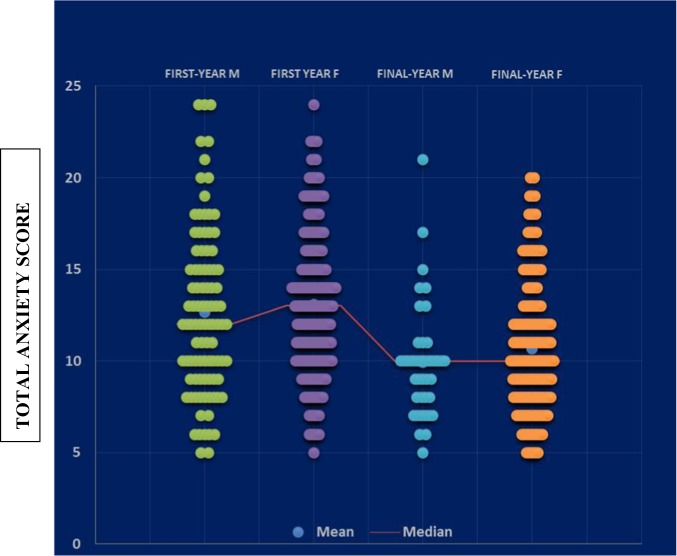
Scatterplots of total anxiety score for first-year male and female, and final-year male and female BDS students. Mean and median values are indicated

Scattergram plots of the total anxiety scores of males and females for both first- and final-year students (Fig. 4) clearly confirmed that there were no significant differences in this particular measure ‘between-genders-within-study-years’ for both the first- and final-year BDS students.

ANOVA analysis of the complete dataset via model 1 demonstrated that there was a very highly significant difference between the two years of study, i.e., first- *vs*. final years (*p* *<* 10^−4^). The ‘between-genders’ and study year × gender interaction components of variation were not statistically significant (*p* *=* 0.073 and 0.807, respectively), the latter indicating that particular combinations of gender and study year do not give rise to a non-additive effect model response. However, the ‘between-genders’ effect was close to statistical significance. Thes ‘between-study year’ difference was also found to be very highly significant when tested by the non-parametric two-tailed Mann–Whitney test (*p* *<* 10^−4^).

Within sub-group analysis of the dataset according to model 2 (ANCOVA) revealed that student age exerted no significant effects in both study year groups (*p* *=* 0.443 and 0.616 for first- and final-year students respectively). Similarly, the gender and the age x gender interaction effects were also not found to be statistically significant for both study years in this model.

As expected, PCR analysis of the standardized raw dataset, which involved the prediction of anxiety scores from student year classifications, and students’ genders and ages, revealed that the best model attained comprised two major significant principal components (PC1 and PC2). PC1 had very strong loadings from first and final course years (0.594 and −0.594 respectively) and age (−0.554), whereas PC2 had strong loadings from gender (−0.844 and 0.844 for males and females respectively). These results confirm that, along with the fully expected strong correlation between student year of study and age, and that gender was independent of (orthogonal to) these predictor variables when total anxiety score served as the dependent variable.

The sources of these study year-mediated differences were found to predominantly arise from the criteria monitored on MDAS scale items 3, 4 and 5 (corresponding to anxiety induced by teeth-drilling, teeth scaling and polishing, and the receiving of local anesthesia respectively), and these are outlined below in Table 2.

**Table 2:** Enumeration distribution of dental anxiety according to year of study, and statistical significance of associations found

Question	Anxiety Response	First-Year (n)	Final-Year (n)	Total (n)	*χ*^2^ *p* Value
If you went to your dentist tomorrow, how would you feel?	Not anxious	93	100	193	0.003
	Slightly anxious	169	118	287	
	Fairly anxious	60	29	89	
	Very anxious	26	12	38	
	Extremely anxious	5	0	5	
If you were sitting in the waiting room, how would you feel?	Not anxious	92	87	179	0.004
	Slightly anxious	140	112	252	
	Fairly anxious	74	44	118	
	Very anxious	32	15	47	
	Extremely anxious	15	1	16	
If you were about to have a tooth drilled, how would you feel?	Not anxious	32	53	85	<0.0001
	Slightly anxious	102	113	215	
	Fairly anxious	81	54	135	
	Very anxious	84	31	115	
	Extremely anxious	50	8	58	
If you were about to have your teeth scaled and polished, how would you feel?	Not anxious	93	156	249	<0.0001
	Slightly anxious	111	79	190	
	Fairly anxious	69	10	79	
	Very anxious	47	12	59	
	Extremely anxious	30	2	32	
If you were about to receive local anesthetic injection in your gum, how would you feel?	Not anxious	42	22	64	0.0004
	Slightly anxious	82	77	159	
	Fairly anxious	70	75	145	
	Very anxious	94	64	158	
	Extremely anxious	64	21	85	

The influence of year of study on student responses to individual MDAS anxiety questionnaire items was explored via *χ*^2^ contingency table analyses (Table 2). These analyses revealed that, with the exception of the final item focused on local anesthesia, final-year BDS students exhibited a much lower level of anxiety than the first-year ones for all questionnaire items on the MDAS questionnaire (*p* *=*  <0.0001 to 0.004). For the question concerning anesthesia, the percentage of first-year students who recorded no signs of anxiety (12%) was approximately two-fold that of final year students; conversely, however, the first-year student group had a *ca*. twice the proportion of students (18%) than those of final-year ones who were extremely anxious about this item. Virtually the same percentages of students in each study year classification were very anxious regarding this criterion (*ca*. 25%).

Notwithstanding, the gender qualitative variable was found to exert no effect whatsoever on anxiety response levels for all of the above MDAS anxiety questions (Table 3).

**Table 3:** Enumeration distribution of level of dental anxiety by gender, and statistical significance of associations found

Question	Anxiety Response	Male (n)	Female (n)	Total (n)	*χ*^2^ *p* Value
If you went to your dentist tomorrow, how would you feel?	Not anxious	148	43	191	ns
	Slightly anxious	219	58	277	
	Fairly anxious	70	19	89	
	Very anxious	28	10	38	
	Extremely anxious	2	2	4	
If you were sitting in the waiting room, how would you feel?	Not anxious	138	38	176	ns
	Slightly anxious	197	55	252	
	Fairly anxious	96	22	118	
	Very anxious	32	14	46	
	Extremely anxious	10	6	16	
If you were about to have a tooth drilled, how would you feel?	Not anxious	67	18	85	ns
	Slightly anxious	163	50	213	
	Fairly anxious	103	32	135	
	Very anxious	92	22	114	
	Extremely anxious	45	12	57	
If you were about to have your teeth scaled and polished, how would you feel?	Not anxious	1	0	1	ns
	Slightly anxious	146	41	187	
	Fairly anxious	60	18	78	
	Very anxious	41	18	59	
	Extremely anxious	25	6	31	
If you were about to receive local anesthetic injection in your gum, how would you feel?	Not anxious	45	19	64	ns
	Slightly anxious	121	37	158	
	Fairly anxious	117	26	143	
	Very anxious	128	30	158	
	Extremely anxious	62	22	84	

Table 4 provides data regarding the numbers of first- and final-year BDS students responding positively or negatively to their perceived oral health status and experiences, and all criteria tested found a series of statistically significant associations between these qualitative enumeration variables and levels of anxiety (*χ*^2^ contingency table analyses). For the student years of study variable, these were the observation of bleeding gums during tooth-brushing (higher in first-year students); the previous performance of dental scaling (much higher in final-year students); the receiving of oral hygiene education (higher in first-year students); and the early choice of dentistry as a career goal (somewhat greater in final-year students, although this was a significantly greater ‘unknown’ selection for first-year students).

**Table 4:** BDS students’ oral health perception responses and statistical significance (χ^2^ *p* values) according to year of study (top) and gender (bottom)

	Group		*X*^*2*^ *p* Value
	First year N (%)	Final year N (%)	
Spontaneously bleeding gums			
Yes	15 (4.9)	8 (3.06)	0.442
No	336 (95.1)	252 (96.5)	
Bleeding gums while brushing?			
Yes	63 (17.8)	26 (9.96)	0.006*
No	288 (81.6)	235 (90.0)	
When do you brush?			
After breakfast	9 (2.5)	6 (2.2)	0.521
Before breakfast	337 (95.4)	245 (93.8)	
No definite time	5 (1.4)	7 (2.6)	
Scaling Done			
Yes	117 (33.1)	227 (86.9)	<0.001*
No	234 (66.2)	31 (11.8)	
Oral hygiene education received			
Yes	257 (72.8)	224 (85.8)	<0.001*
No	91 (25.7)	32 (12.2)	
Was dentistry your preferred career goal?			
Yes	292 (82.7)	234 (89.6)	0.007*
No	20 (5.6)	12 (4.5)	
Don’t Know	35 (9.9)	9 (3.4)	

	Gender		*X*^*2*^ *p* Value
	Female N (%)	Male N (%)	
Spontaneously bleeding gums			
Yes	19 (3.9)	4 (2.9)	0.593
No	455 (95.1)	129 (94.8)	
Bleeding gums while brushing?			
Yes	66 (13.8)	22 (16.1)	0.002*
No	409 (85.5)	111 (81.6)	
When do you brush?			
After breakfast	8 (1.6)	7 (5.1)	<0.001*
Before breakfast	453 (94.7)	119 (87.5)	
No definite time	6 (1.2)	10 (7.3)	
Scaling Done			
Yes	267 (55.8)	66 (48.5)	<0.001*
No	10 (2.0)	70 (51.4)	
Oral hygiene education received			
No	86 (17.9)	41 (30.1)	<0.001*
Yes	383 (80.1)	95 (69.8)	
Yes	77 (16.0)	29 (21.3)	
Was dentistry your preferred career goal?			
Yes	407 (85.1)	112 (23.4)	0.423
No	28 (5.8)	8 (5.8)	
Don’t Know	30 (6.2)	13 (9.5)	

Statistically significant considerations found ‘between-genders’ were the identification of bleeding gums whilst tooth-brushing (higher in males); time of tooth-brushing (significantly more for males when this regimen is performed after breakfast); previous experience of dental scaling (much higher in females); and the reception of oral health education (significantly greater in females).

Table 5 shows that there was a statistically significant difference in the total Level of dental anxiety (*p* *<* 0.001) between first and final year students, but no significant difference was detected for Body mass index (BMI) (*p* *>* 0.05) between the two BDS degree student groups. However, Table 6 shows that there was a significant between-gender difference in BMI values, with males having a higher mean value than that of females (*p* < 0.001). However, no correlations were observed between the total dental anxiety level and BMI values.

**Table 5:** Age, BMI and Anxiety Score among students in each study group

	First year		Final year		*p* Value
	Mean	SD	Mean	SD	
Age	18.31	1.08	21.54	0.95	<0.001*
BMI	22.09	4.01	21.63	3.84	0.182
Anxiety score	12.96	4.00	10.52	3.40	<0.001*

**Table 6:** Age, BMI and Anxiety Score by gender in each study group

	Female		Male		*t* test value	*p* Value
	Mean	SD	Mean	SD		
Age	19.69	1.89	19.68	2.00	0.06	0.953
BMI	21.58	3.77	23.08	4.33	3.65	<0.001*
Anxiety score	11.98	3.84	11.67	4.16	0.14	0.887

## Discussion

### Dental anxiety

In this study, the status of dental anxiety was monitored using the MDAS scale, and related information was collected by using a pre-tested, validated questionnaire. The level of dental anxiety was higher amongst first-year BDS students over those of final-year ones (Fig. 1). These results are consistent with previous studies performed elsewhere.^10,18,22,29–32^ A probable reason for this is that final-year students are more acclimatized with their clinical settings and scenarios. Dental anxiety is a cause of poor patient (in this case dental student) compliance and negative attitudes towards receiving dental treatment by the sufferers. Indeed, dentists find it very difficult to treat anxious patients, who may be further afflicted by a series of oral diseases and oral health problems, since they delay or even terminate visits to dental surgeries. Notwithstanding, dental health education strategies and acclimatization to dental procedures can facilitate reductions in stress levels; similar strategies could be utilized for anxious dental students, in order to assist them in tackling their dental anxiety problems.^33,34^

In view of the above observations, and the strong positive correlation of age with students’ year of study, the mean anxiety score also decreased with increasing age, as expected and noted in.^22^ However, from our ANCOVA model 2 analysis performed, no significant differences were found between ages ‘nested’ within study year classification groups.

It was also observed that there was an increased level of anxiety in all the categories of the MDAS score system amongst first- and final-year dental students. Specifically, first-year students were more anxious with MDAS items 3, 4 and 5 when compared to the corresponding questionnaire responses of final year ones (*p* < 0.001) (Table 2). The possible reason for this is that, final year students have more exposure to treatment procedures, and this experience assists the process of dental treatment acclimatization. Indeed, the administration of local anesthetics and the drilling of teeth were associated with a higher level of anxiety. Indeed, 26.6% of first-year students replied that they are very anxious during the drilling of teeth, and 18.1% confirmed that they were extremely anxious about receiving a local anesthetic injection; this observation is in agreement with previous studies documented elsewhere.^35,36^ Approximately one-quarter (24%) of students were very anxious, and 14% were extremely anxious regarding the prospect of receiving dental treatment, and these experiences usually began whilst these subjects are awaiting such treatment in dental surgery reception areas. Their responses revealed that the drilling of a carious tooth was a predominant cause of dental anxiety, and this finding is also similar to that found in previous study reports.^35,36^

An improved knowledge of painless dental procedures, along with education for oral hygiene maintenance through consistent and personalized approaches, may assist patients to overcome at least some forms of dental apprehension. Indeed, a fully structured tutor-led awareness programme from the very start of dental undergraduate courses may serve as the best approach for intervention. Dental anxiety can also be effectively managed at an earlier stage by the enhancement of levels of awareness through the introduction of a motivational programme at both school and pre-university levels. Awareness of the positive benefits of dental treatment, delivered and learnt at an early stage in life, may facilitate reductions in dental anxiety responses. Since generic approaches to such issues may be ineffective, a more personalized strategy may be effective in diminishing the level of dental anxiety significantly.^33^ However, in order to alleviate distressing dental anxiety experiences long-term, a time-framed follow-up with a sensitization process employing empowered reinforcement may be required.

### Oral health

The survey featuring a questionnaire on periodontal health showed that oral health status was improved amongst final year students over that of first year ones. A total of 26 (9.96%) students in the final year explained that they experienced bleeding on brushing, compared to 63 students (17.8%) experienced by those in the first year of their BDS course (Table 4). This study also found that there was a significant association between dental anxiety levels and oral hygiene in first year students, and more commonly in females. These results concur with those of a further study.^36^ Since the co-existence of dental anxiety and poor oral health-related quality of life (QoL) is not simply explicable, further investigations are required to explore this. However, both oral health-related QoL and dental anxiety are inter-dependent phenomena, the former enhancing the latter’s levels.^36^

The frequency of dental visits for oral prophylaxis was higher among final-year BDS students than it was in first-year ones: this is especially evident among female students. This study also demonstrated that a higher number of first-year students received oral hygiene education than final year ones, and hence despite this deficit, final-year students had the scope required to apply their learning based on clinical resources (such as patients based at hospital and community settings). This is undoubtedly reinforced thorough their observations experienced during clinical placements.

Therefore, early implementation and reinforced dental awareness, together with the implementation of an appropriate acclimatization process, can give rise to a significant reduction in dental anxiety; this has been observed in related studies.^2,15^

### Dentistry as a career and dental anxiety

The majority of the dental students involved in this study revealed that they had chosen dentistry as a career choice of their own accord. However, a small proportion of female students (5%) did not choose the dental profession as their first career of choice (Table 3). In this study we could not establish an inter-relationship between dental anxiety and likelihood of dentistry as a choice of career. Moreover, to date, there are no published reports available which have investigated whether or not dental anxiety affects or influences students’ choice of dentistry as a future career prospect.

## Clinical implications

Oral health awareness, empathetic attitudes towards patients, sensitization, and acclimatization of treatment procedures are required to be reinforced for first-year BDS students. This will not only facilitate reductions in their levels of dental anxiety experienced individually, but also such individual experiences and sufferings could promote the development of realistic strategies for tackling anxious patients when practicing as a dentist following completion of their studies.

## Conclusions

This study provided a high level of evidence that first-year dental students experience a moderately higher level of dental anxiety than final-year ones. But, the MDAS scores are lower than the threshold level (i.e., ≥ 19 MDAS Score). Therefore, there is a major requirement to further investigate such anxiety amongst medical students in order to compare their profiles with those studying dentistry.

Local anesthesia injections and drilling of teeth significantly elevates anxiety levels in both first year and final-year BDS students. However, an adequate explanation of measures for painless delivery of these anesthetics may assist in attenuating this level of dental anxiety. Moreover, behavioral therapies and distraction methods may also be effective in this context. Therefore, there is a major requirement for further investigations of the nature and level of anxiety amongst medical students in order to compare their anxiety profiles with those studying dentistry.
